# Effect of Pulsed Electric Fields on the Flavour Profile of Red-Fleshed Sweet Cherries (*Prunus avium var.* Stella)

**DOI:** 10.3390/molecules20035223

**Published:** 2015-03-23

**Authors:** Kristine Ann Gualberto Sotelo, Nazimah Hamid, Indrawati Oey, Noemi Gutierrez-Maddox, Qianli Ma, Sze Ying Leong

**Affiliations:** 1 School of Applied Sciences, Faculty of Health and Environmental Sciences, Auckland University of Technology, Auckland 1142, Private Bag 92006, New Zealand; E-Mails: kristine.sotelo_aut@yahoo.co.nz (K.A.G.S.); noemi.gutierrezmaddox@aut.ac.nz (N.G.-M.); roy.ma@aut.ac.nz (Q.M.); 2 Department of Food Science, University of Otago, PO Box 56, Dunedin 9054, New Zealand; E-Mails: indrawati.oey@otago.ac.nz (I.O.); sze.leong@otago.ac.nz (S.Y.L.)

**Keywords:** volatile compounds, SPME, GC-MS, sweet cherries, pulsed electric fields

## Abstract

The aim of this research was to study the effect of pulsed electric fields (PEF) on the flavour profile of red-fleshed sweet cherries (*Prunus avium variety* Stella). The cherry samples were treated at a constant pulse frequency of 100 Hz, a constant pulse width of 20 μs, different electric field strengths between 0.3 and 2.5 kV/cm and specific energy ranging from 31 to 55 kJ/kg. Volatile compounds of samples were analysed using an automated headspace solid phase microextraction (HS–SPME) method coupled with gas chromatography-mass spectrometry (GC–MS). A total of 33 volatile compounds were identified with benzaldehyde, hexanal, (*E*)-2-hexenal, (*Z*)-2-hexen-1-ol, and benzyl alcohol being the predominant volatiles in different PEF-treated samples. Aldehydes namely butanal, octanal, 2-octenal, and nonanal, and (*Z*)-2-hexen-1-ol increased significantly 24 h after PEF treatment at electric field strengths of more than 1.0 kV/cm. Samples incubated for 24 h after PEF treatment (S3) generated higher concentrations of volatiles than samples immediately after PEF treatments (S2). Quantitative results revealed that more flavour volatiles were released and associated with S3 samples after 24 h storage and S2 samples immediately after PEF both with the highest electric field intensities. Interestingly, this study found that the PEF treatments at the applied electric field strength and energy did not result in releasing/producing undesirable flavour compounds.

## 1. Introduction

Sweet cherry (*Prunus avium* L.) fruit is one of the most popular temperate fruits [1]. Globally, only 7% of sweet cherries are grown in the southern hemisphere, where New Zealand, Australia, and Chile are the world’s biggest exporters [2]. Due to the relatively short growing season, a large part of the cherry production is processed as brined, canned, frozen and dried fruits or made into juices or syrups [1]. Frozen fruit are commonly used as intermediate raw material for a variety of food products including jams, preserves, and smoothies [3].

Consumers like sweet cherries because of their sweetness and wealth of nutrients. However, processed cherry fruits require strict treatment conditions to protect their quality, especially flavour, which is an important attribute in determining consumers’ acceptability [4]. Sweet cherries contain a great number of volatile compounds, wherein the major constituents are aldehydes, alcohols, acids, esters, alkanes, aromatic hydrocarbons, ketones, and terpenes. Several researchers have studied the flavour volatiles of cherries. According to Schmid and Grosch [5], volatiles such as benzaldehyde, (*E*)-2-hexenal, and hexanal were reported to be the major flavour compounds found in cherries. Mattheis [6] reported that a total of 28 aroma compounds were detected in “Bing” sweet cherry fruit using headspace sampling wherein 2-propanol and ethanol had relatively high concentrations while benzaldehyde and hexanal had high aroma values and were highly correlated with fresh cherry aroma. In the study of Girard and Kopp [7], 50 volatile compounds were identified in 12 sweet cherries using a dynamic headspace method coupled with GC-MS. They reported that (*E*)-2-hexenol, benzaldehyde, hexanal and (*E*)-2-hexenal were the predominant flavour volatiles in sweet cherries. The same technique was used by Bernalte *et al.* [8] who identified 62 volatile compounds in sweet cherry. Of these, hexanal and (*E*)-2-hexenal were the major compounds found. Zhang and co-workers [9] studied the aroma components of “Hongdeng” sweet cherry using HS-SPME, followed by GC-MS which led to the identification of 37 compounds. Six compounds, including hexanal, (*E*)-2-hexenal, benzaldehyde, (*E*)-2-hexen-1-ol, ethyl acetate, and hexanoic acid ethyl ester were found to be characteristic aroma components of sweet cherry fruit.

Pulsed electric field (PEF) processing is a non-thermal food processing technology based on the application of short pulses of high voltage through a food product, whether in semi-solid or liquid form, which is placed between two electrodes [10]. This process is carried out at either low or moderate temperature and is a promising non-thermal extraction technique [11]. The effect of low or moderate intensity PEF has been mainly studied on the extraction of bioactive compounds from fruits and vegetables. PEF has been reported to be highly effective method for extraction of intracellular compounds. The application of PEF on food induces cell membrane permeabilization through a phenomenon called “electroporation” [12]. This electropermeabilization refers to the ability of PEF to induce pore formation within cellular components due to the localised structural changes and the breakdown of the cellular membrane [10] that in turn, allows the extraction of plant compounds. Pore formation can be reversible or irreversible process depending on the intensity of electric fields being applied [10]. Toepfl [13] reported that the extractability of fruit and vegetable juices or intracellular compounds can be enhanced after PEF-treatment. Higher juice yield (5% more) with higher total anthocyanin content (three times higher) was obtained after PEF treatment at 3 kV/cm applied to grape samples [14]. Higher concentration of bioactive compounds such as polyphenols in tomatoes was found after PEF treatment [15]. Similar finding was also found that PEF treatment at 0.5 kV/cm increased the total polyphenolic (TP) content in fresh pressed grape juice by 13% in comparison to reference sample [16]. The amount of isoflavonoid diadzein in soybean increased by 20% after PEF treatment at 1.3 kV/cm [17].

To date, the effect of PEF treatment at low or moderate intensity on the flavour profile of fruit and vegetables has not been studied much. Vallverdú-Queralt *et al.* [18]. indicated that PEF treatment at moderate intensity increased the concentration of hexanal and (*E*)-2-hexenal flavour compounds in tomatoes processed into juice. The effect of PEF treatment on the volatile profiles has been mostly studied at the high PEF treatment intensity required for preservation (28 to 40 kV/cm electric field strengths). Those studies showed that PEF processing changes the flavour volatiles of watermelon juice during storage [19], strawberry juices [20], citrus juices [21], apple juice [22], longan juice [23], and orange juices [24,25,26,27].

Therefore, the objective of this research was to study the effect of low or moderate PEF treatment at different electric field strengths on the flavour profile of red-fleshed sweet cherries (*Prunus avium variety* Stella) in order to produce intermediate natural smoothie-like products, with enhanced flavour qualities. Stella cultivar is described as a large, heart-shaped fruit, red to dark red in colour, and sweet flavoured cherry. The profile of flavour compounds immediately after PEF treatment was compared with that after 24 h.

## 2. Results and Discussion

### 2.1. Identification of Volatile Compounds

The effect of PEF treatments and storage conditions on cherry samples is shown in Table 1. A total of 33 compounds were identified in all cherry samples. Most of the compounds have been reported previously to be characteristic of sweet cherry flavour from previous studies [28]. These volatiles largely comprised 15 aldehydes, 10 alcohols, four esters, two terpenes, one acid and one alkane. Aldehydes and alcohols were the dominant volatile groups in all cherry samples, followed by alkane, esters, terpenes, and acid. In the aldehyde and alcohol groups, (*E*)-2-hexenal, benzaldehyde, hexanal, (*Z*)-2-hexen-1-ol, and benzyl alcohol were among the highest. These results were similar to other researches [7,9,29]. However, application of PEF treatments significantly increased the volatile concentrations of hexanal, (*E*)-2-hexenal, benzaldehyde, (*Z*)-2-hexen-1-ol and benzyl alcohol (Table 1). C_6_ compounds and aromatic compounds were among the most significant class of compounds according to their ratio to the internal standard, *i.e.*, hexanal (36.65–209.69), (*E*)-2-hexenal, (129.91–1109.53), benzaldehyde (49.33–882.79), (*Z*)-2-hexen-1-ol (2.69–26.09), and benzyl alcohol (17.46–129.45). 

**Table 1:** Volatile compounds in control and PEF-treated cherries (immediately after PEF and 24 h after PEF).

No.	Volatile Compounds	RI ^α^	Identification ^β^	Treatment *															
				S1 (Control)	S2P1	S2P2	S2P3	S2P4	S2P5	S2P6	S2P7	S3P0	S3P1	S3P2	S3P3	S3P4	S3P5	S3P6	S3P7
*Aldehydes*																			
1	Butanal	<653	MS	0.08 ^b^	ND	0.40 ^ab^	0.23 ^ab^	0.34 ^ab^	0.30 ^ab^	0.30 ^ab^	ND	ND	ND	0.34 ^ab^	0.32 ^ab^	1.00 ^a^	1.00 ^a^	ND	0.09 ^b^
2	Pentanal	666	MS+RI	0.86 ^abc^	ND	1.14 ^abc^	0.52 ^abc^	0.71 ^abc^	0.46 ^bc^	0.73 ^abc^	1.69 ^a^	0.30 ^c^	1.48 ^ab^	ND	0.21 ^c^	ND	0.02 ^c^	ND	ND
3	2-Pentenal, (E)-	756	MS+RI	ND	ND	ND	0.04 ^b^	0.01 ^b^	0.14 ^ab^	ND	ND	ND	0.06 ^b^	0.05 ^b^	ND	ND	0.20 ^a^	0.06 ^b^	ND
4	Hexanal	804	MS 85%	50.91 ^b^	87.29 ^b^	134.60 ^ab^	73.97 ^b^	149.37 ^ab^	119.94 ^ab^	83.04 ^b^	81.91 ^b^	36.65 ^b^	85.12 ^b^	123.48 ^ab^	55.51 ^b^	209.69 ^a^	74.75 ^b^	104.66 ^ab^	117.46 ^ab^
5	2-Hexenal, (E)-	858	MS 85%	310.92 ^bc^	243.13 ^bc^	613.11 ^abc^	253.49 ^bc^	1109.53 ^a^	585.49 ^abc^	321.77 ^bc^	282.74 ^bc^	129.91 ^c^	402.29 ^bc^	646.29 ^abc^	176.06 ^bc^	672.14 ^ab^	295.66 ^bc^	593.69 ^abc^	521.42 ^bc^
6	Heptanal	906	MS+RI	0.19 ^c^	0.59 ^bc^	1.24 ^bc^	0.50 ^bc^	0.77 ^bc^	1.02 ^bc^	2.73 ^a^	1.23 ^bc^	0.51 ^bc^	0.82 ^bc^	1.26 ^bc^	0.99 ^bc^	1.32 ^bc^	1.55 ^ab^	1.31 ^bc^	1.59 ^ab^
7	2,4-Hexadienal, (E,E)-	927	MS 85%	3.29 ^abc^	2.32 ^abc^	4.35 ^abc^	2.95 ^abc^	6.20 ^a^	5.61 ^ab^	0.53 ^c^	2.89 ^abc^	1.78 ^bc^	ND	5.53 ^ab^	3.72 ^abc^	5.71 ^a^	3.50 ^abc^	5.97 ^a^	4.42 ^abc^
8	2-Heptenal, (Z)-	963	MS+RI	0.07 ^b^	ND	ND	0.18 ^b^	0.01 ^b^	0.04 ^b^	ND	0.17 ^b^	ND	ND	ND	ND	0.47 ^b^	0.28 ^b^	1.90 ^a^	0.17 ^b^
9	Benzaldehyde	968	MS 85%	166.78 ^bc^	125.02 ^bc^	153.17 ^bc^	108.97 ^bc^	179.31 ^bc^	154.14 ^bc^	363.65 ^b^	882.79 ^a^	49.33 ^c^	102.60 ^bc^	140.80 ^bc^	65.86 ^c^	192.09 ^bc^	66.73 ^c^	184.00 ^bc^	114.72 ^bc^
10	2,4-Nonadienal, (E,E)-	992	MS 85%	ND	ND	ND	0.19 ^b^	ND	ND	ND	ND	0.11 ^b^	ND	ND	ND	1.25 ^b^	3.28 ^a^	ND	0.24 ^b^
11	Octanal	1007	MS 85%	0.30 ^b^	0.37 ^b^	0.22 ^b^	0.29 ^b^	0.23 ^b^	0.74 ^b^	ND	1.36 ^b^	0.68 ^b^	1.06 ^b^	0.60 ^b^	5.20 ^a^	1.02 ^b^	1.39 ^b^	0.84 ^b^	0.70 ^b^
12	2-Octenal, (E)-	1034	MS 85%	0.61 ^c^	ND	0.75 ^bc^	0.41 ^c^	0.14 ^c^	ND	0.90 ^bc^	0.81 ^bc^	1.33 ^bc^	ND	ND	1.76 ^abc^	4.41 ^a^	3.43 ^ab^	2.97 ^abc^	1.18 ^bc^
13	Nonanal	1109	MS 85%	0.88 ^b^	1.69 ^b^	3.17 ^b^	ND	ND	2.18 ^b^	ND	6.10 ^b^	3.32 ^b^	4.47 ^b^	2.95 ^b^	16.10 ^a^	3.45 ^b^	3.70 ^b^	4.79 ^b^	4.88 ^b^
14	2-Nonenal, (E)-	1166	MS+RI	0.39 ^b^	ND	0.02 ^b^	ND	ND	0.88 ^a^	ND	ND	0.31 ^b^	ND	0.29 ^b^	0.24 ^b^	ND	0.02 b	1.25 ^a^	ND
15	Decanal	1211	MS 85%	0.22 ^c^	0.41 ^bc^	1.50 ^ab^	0.16 ^c^	0.29 ^bc^	0.56 ^abc^	ND	1.28 ^abc^	0.42 ^bc^	1.83 ^a^	0.34 ^bc^	0.37 ^bc^	0.74 ^abc^	0.94 ^abc^	0.60 ^abc^	0.66 ^abc^
*Alcohols*																			
16	3-Pentanol	*<653*	MS	3.56 ^a^	ND	ND	ND	ND	ND	ND	ND	0.27 ^b^	ND	ND	ND	ND	ND	ND	ND
17	1-Penten-3-ol	661	MS+RI	0.34 ^b^	ND	ND	ND	ND	ND	0.82 ^b^	ND	ND	ND	ND	0.23 ^b^	ND	2.24 ^a^	0.48 ^b^	0.45 ^b^
18	2-Hepten-1-ol, (Z)-	701	MS+RI	0.05 ^d^	0.24 ^c^	0.99 ^ab^	0.36 ^bc^	0.18 ^c^	0.60 ^bc^	0.77 ^bc^	1.52 ^a^	ND	0.17 ^c^	ND	0.32 ^bc^	ND	0.16 ^c^	0.16 ^c^	0.42 ^bc^
19	3-Buten-1-ol, 3-methyl-	733	MS+RI	0.64 ^b^	0.67 ^b^	1.51 ^ab^	0.65 ^a^	1.22 ^ab^	1.05 ^b^	0.66 ^b^	2.45 ^a^	0.44 ^b^	ND	0.48 ^b^	0.34 ^b^	0.69 ^b^	0.75 ^b^	1.00 ^b^	0.75 ^b^
20	2-Hexen-1-ol, (Z)-	871	MS+RI	2.69 ^d^	11.45 ^bcd^	11.68 ^abcd^	16.72 ^abcd^	26.09 ^a^	ND	7.58 ^cd^	22.62 ^abc^	12.27 ^a^	11.05 ^bcd^	20.57 ^a^	15.17 ^a^	22.52 ^a^	13.26 ^d^	24.49 ^ab^	11.47 ^bcd^
21	2-Buten-1-ol, 3-methyl-	781	MS+RI	0.69 ^abc^	0.56 ^abc^	0.92 ^abc^	0.65 ^abc^	1.29 ^a^	1.05 ^ab^	0.89 ^abc^	0.60 ^abc^	0.50 ^bc^	ND	0.76 ^abc^	0.34 ^ab^	0.69 ^ab^	0.31 ^c^	1.23 ^ab^	0.73 ^abc^
22	2-Nonen-1-ol, (E)-	947	MS+RI	0.01 ^b^	ND	ND	ND	ND	ND	2.77 ^a^	ND	ND	ND	ND	0.16 ^b^	ND	ND	ND	ND
23	3-Heptanol	957	MS+RI	0.15 ^b^	0.68 ^b^	0.67 ^b^	0.37 ^b^	0.52 ^b^	1.08 ^b^	ND	2.55 ^a^	0.16 ^b^	0.20 ^b^	0.42 ^b^	1.16 ^b^	0.51 ^b^	ND	ND	0.41 ^b^
24	Benzyl Alcohol	1044	MS 85%	59.17 ^bc^	46.17 ^bc^	32.94 ^bc^	27.03 ^bc^	95.72 ^ab^	72.82 ^abc^	52.02 ^bc^	129.45 ^a^	22.74 ^c^	46.22 ^bc^	43.67 ^bc^	17.46 ^c^	65.15 ^abc^	25.94 ^c^	78.42 ^abc^	44.50 ^bc^
25	2-Hexadecanol	1401	MS+RI	0.07 ^b^	0.19 ^ab^	0.74 ^ab^	0.18 ^ab^	0.02 ^b^	0.18 ^ab^	1.46 ^ab^	0.79 ^ab^	1.18 ^ab^	0.15 ^ab^	ND	ND	0.55 ^ab^	1.08 ^ab^	0.20 ^ab^	1.75 ^a^
*Esters*																			
26	Butanoic acid, methyl ester	723	MS+RI	0.03 ^b^	0.06 ^b^	0.30 ^b^	0.20 ^b^	0.20 ^b^	0.14 ^b^	ND	ND	0.14 ^b^	2.99 ^a^	0.19 ^b^	0.08 ^b^	1.28 ^b^	0.25 ^b^	1.29 ^b^	0.16 ^b^
27	Acetic acid, 2-phenylethyl ester	916	MS+RI	ND	ND	0.91 ^a^	ND	ND	0.68 ^a^	ND	ND	ND	0.67 ^a^	ND	ND	ND	ND	ND	ND
28	Benzoic acid, 2-hydroxy-, methyl ester	1201	MS+RI	0.46 ^b^	ND	0.74 ^b^	0.72 ^b^	2.52 ^b^	ND	ND	ND	0.56 ^b^	ND	1.25 ^b^	22.25 ^a^	1.82 ^b^	ND	1.62 ^b^	ND
29	Decanoic acid, methyl ester	1326	MS+RI	3.31 ^b^	ND	ND	ND	ND	ND	ND	ND	ND	ND	ND	15.58 ^a^	ND	ND	ND	ND
*Terpenes*																			
30	Menthol	1186	MS 85%	0.05 ^b^	ND	ND	ND	ND	ND	11.26 ^a^	ND	ND	ND	ND	ND	ND	0.21 ^b^	0.19 ^b^	0.13 ^b^
31	Geranyl vinyl ether	1259	MS 85%	0.25 ^ab^	0.36 ^ab^	0.80 ^ab^	0.34 ^ab^	0.75 ^ab^	0.67 ^ab^	0.12 ^b^	0.45 ^ab^	0.29 ^ab^	ND	0.74 ^ab^	ND	0.90 ^a^	ND	0.66 ^ab^	0.60 ^b^
*Acids*																			
32	n-Decanoic acid	1312	MS 85%	0.02 ^b^	ND	ND	ND	ND	0.30 ^ab^	ND	0.04 ^b^	0.10 ^b^	ND	ND	ND	0.58 ^a^	ND	0.25 ^b^	0.13 ^b^
*Alkanes*																			
33	Undecane	1101	MS 85%	4.69 ^ab^	2.90 ^b^	6.13 ^ab^	4.90 ^ab^	6.86 ^ab^	5.81 ^ab^	ND	4.65 ^ab^	ND	ND	6.57 ^ab^	10.72 ^ab^	6.18 ^ab^	13.10 ^a^	7.49 ^ab^	5.02 ^ab^

^a,b,c^ Different letters within the same row (different treatment for the same volatile compounds) differ significantly using Fisher’s least significant difference (*p* < 0.05). * Ratio to internal standard; S1 = control, S2 = PEF, S3 = 24 h incubation at 4 °C after PEF P 0, 1, 2, 3, 4, 5, 6, 7 = 0, 0.3, 0.7, 1.0, 1.4, 1.7, 2.1, 2.5 kV·cm^−1^. ND: not detected. ^α^ RI on a VF-5MS column, was calculated in relation to the retention time of *n*-alkane (C7–C30) series. ^β^ MS, tentative identification by comparison of mass spectrum with NIST library spectrum (over 85%); MS+RI, mass spectrum identified using NIST mass spectral database and RI agree with literature values [29].

According to Sun and others [29] and Zhang and others [9], the highest content of hexanal and (*E*)-2-hexenal were present in the Stella cultivar with relative peak areas of 21.0 and 6.81, respectively. These values were very low compared to the values obtained after the cherries were processed for both samples immediately after PEF and 24 h after PEF (Table 1).

The C_6_ aldehydes and alcohols are generated by the consecutive action of the enzymes lipoxygenase and alcohol dehydrogenase on polyunsaturated fatty acids [30]. In the present study, it was found that production of C_6_ aldehydes and alcohols was enhanced by the electroporation effects of PEF application. Studies have reported that C_6_ aldehydes are produced from enzymatic reactions, which are attributed to the quantities of precursor molecules already present in the fruit [8,28]. Hexanal and (*E*)-2-hexenal are products of fatty acid oxidation (linoleic and linolenic acid oxidation) in the presence of lipoxygenase, while (*Z*)-2-hexen-1-ol is a secondary compound from these oxidation reactions [31]. Benzaldehyde is produced from the hydrolysis of amygdalin present in cherries [9]. Fatty acid and lipid oxidation reactions can also be attributed to the mechanism of electropermeabilisation by PEF. When an external electric field is applied on biological cells (animal, plant or microbial), disruption of the cell membrane occurs [32].This results in the increase of enzymes that react to produce and increase the volatile compounds found in this study.

The C_6_ compounds are known to have desirable odours. Hexanal and (*E*)-2-hexenal have a characteristic green leaf-odour, while benzaldehyde, benzyl alcohol, and (*Z*)-2-hexen-1-ol are described as having almond-like, floral, and vegetable odour, respectively [29,33]. These important volatiles have also been reported by Sun and others [29] to have high flavour dilution factors. According to them, the significant aroma compounds that greatly contribute to the aroma profile of Stella cultivar that had the highest flavour dilution value of >64 were hexanal, (*E*)-2-hexenal, following were (*Z*)-3-hexenal and benzaldehyde which had 32 FD values, while octanal, 2–4-nonadienal, and (*Z*)-3-hexen-1-ol had an FD value of 16.

A significant increase in the level of benzaldehyde was found after the application of 2.5 kV/cm (S2P7) immediately after PEF. This was probably due to the electropermeabilization effect of the high electric field strength, which increased and stimulated the metabolic activity of enzymes present in the plant cell [15]. No significant differences were found between samples except for the S2P7. Majority of the samples after 24 h decreased compared to S2 samples immediately after PEF, but the decrease was not significant from the control. Aldehydes namely butanal, octanal, (*E*)-2-octenal, and nonanal with concentrations between 0.01 to 16.1 increased significantly after 24 h of incubation except for pentanal, which decreased after 24 h of incubation. Aldehydes such as (*E*)-2-pentenal, (*Z*)-2-heptenal, (*E,E*)-2,4-nonadienal, 2-nonenal were noticeably not present in most of the samples immediately after PEF but were released after 24 h of incubation. Two of the S3 samples (S3P4 and S3P5) had a higher concentration of (*E,E*)-2, 4-nonadienal compared to a previous study of Sun *et al.* [29]. The compound (*E,E*)-2,4-nonadienal, which has a fatty odour, was determined to be one of the aroma-active compounds that greatly contributed to the aroma of Stella cultivar [29]. The concentration of hexanal increased after PEF treatments both immediately after PEF and 24 h after PEF. At 1.4, 2.1, and 2.5 kV/cm electric field strengths an increase in concentration was observed after 24 h but the increase was only significant in S3P4 samples at 1.4 kV/cm electric field strength. The concentration of heptanal increased after PEF treatments. Sample S2P6 with 2.1 kV/cm electric field strength was observed to be significantly different in heptanal among S2 samples.

(*Z*)-2-Hexen-1-ol was detected in all samples except for the S2P5 sample. There was a significant increase (*p* < 0.05) in the concentration of (*Z*)-2-hexen-1-ol for S2 (S2P4 and S2P7) and most S3 (S3P2, S3P3, S3P4, and S3P6) samples compared to control sample, immediately after PEF and after 24 h incubation. Benzyl alcohol was present in the majority of samples immediately after PEF. However, it was only significantly higher in S2P7 (2.5 kV/cm electric field strength) after 24 h. 

### 2.2. Volatile Analysis of Cherry Samples Untreated and Treated with Different PEF Energy Intensities

#### Headspace Volatile Compounds of PEF-Treated Cherries

Based on the data in Table 1, PEF samples treated with the low PEF intensity produced higher concentrations of volatile compounds. This is in agreement with the findings of Toepfl and others [13] who studied the permeabilization of cell membranes in food by PEF reported that low intensity PEF can potentially induce stress reactions in plant systems or cell cultures, so that bioproduction of certain compounds are enhanced and stimulated. As seen in Table 1, more flavour volatiles were released in S3 samples after 24 h storage, and S2 samples immediately after PEF treatment at the highest electric field intensities. These samples were S3P4, S3P5, S3P6, S3P7, S2P5, S2P6, and S2P7 with electric field strengths of 1.40 to 2.50 kV/cm, and specific energy input of 30.0 to 47.0 kJ/kg. 

A total of fourteen volatile compounds were associated with S3 samples (S3P4, S3P5, S3P6, S3P7 with electric field strength of 1.4, 1.7, 2.1, 2.5 kV/cm respectively) with the highest concentrations being mostly of aldehydes such as butanal, hexanal, (*E*)-2-hexenal, heptanal, (*E*,*E*)-2,4-hexadienal, (*E*)-2-pentenal, (*E*)-2-octenal, (Z)-2-heptenal, decanal; three alcohols such as 1-penten-3-ol, 2-hexadecanol, 3-methyl-3-buten-1-ol, one terpene—geranyl vinyl ether—and one acid—*n*-decanoic acid. Most of these volatiles have been reported in previous studies on cherries [29,34]. Green and grassy were the most intense odour characteristic of cherries. This attribute, as mentioned earlier, is due to the C_6_ aldehydes (hexanal and (*E*)-2-hexenal). (*E*,*E*)-2,4-Hexadienal also produces a sweet, green, and fruity aroma in cherries. 2-Octenal was responsible for the green and nutty flavour, while 2-heptanal has been described as almond-like in odour [29,34]. The compound decanal has been reported to have citrusy, orange, and green attributes [29,34]. However, according to Wen *et al.* [34], decanal has not previously been described as an important aroma compound in sweet cherries.

S2 samples namely S2P5, S2P6, S2P7 having electric field strength of 1.7, 2.1, and 2.5 kV/cm respectively have the highest concentrations of aldehydes namely, (*E*)-2-nonenaland benzaldehyde; alcohols such as (*E*)-2-nonen-1-ol, 3-methyl-2-buten-1-ol, (*Z*)-2-hepten-1-ol, (*Z*)-2-hexen-1-ol, and benzyl alcohol; esters such as butanoic acid methyl ester and acetic acid, 2-phenylethyl ester; and one terpene, which is menthol. It is worth noticing that samples immediately after PEF treatment with higher energy intensities produced the highest level of benzaldehyde and benzyl alcohol. Benzaldehyde is said to be the primary contributor to the characteristic flavour of sweet cherry fruit and benzyl alcohol is its deramification product [9]. Benzaldehyde and benzyl alcohol are responsible for the almond and floral odour in cherries [29,34] while it is also reported that (*E*)-2-nonenal and (*E,Z*)-2,6-nonadienal impart a green attribute described as fresh cucumber. Similarly, green and vegetable attributes may be due to (*Z*)-2-hexen-1-ol, while minty attributes may originate from menthol [29].

Two S3 samples namely S3P2 and S3P3 treated with 0.7 and 1.0 kV/cm electric field strength respectively resulted in the highest concentrations of a total of eight volatile compounds, namely nonanal, octanal, 2-hydroxybenzoic acid methyl ester, decanoic acid methyl ester, 3-heptanol, and undecane. Nonanal contributed to the citrusy (orange-like) and green aroma in cherries while octanal was reported to have a fruity (lemon-like) and green aroma as well. [29,34].

Finally, control (S1 and S3P0) and PEF treated samples (S2 samples: S2P1, S2P2, S2P3, S2P4; S3 sample: S3P1) with the lowest energy intensities (S2 samples: 0.3, 0.7, 1.0, and 1.4 kV/cm respectively; S3 sample: 0.3 kV/cm) had the least volatiles. However, these samples had the highest concentrations of pentanal and 3-pentanol. The impact of electroporation on the samples 24 h after PEF treatment resulted in significantly increased concentrations of most of the aldehydes. This suggested that volatile compounds after PEF processing required time to be released. When low or moderate PEF is applied, electropermeabisation occurs, which creates reversible process of pore formation. Through this process, volatile compounds are released following the reactions of lipoxygenase enzymes. Guderjan, Töepfl, Angersbach and Knorr [17] reported that after the application of low intensity PEF (0.6 kV/cm electric field strength and 0.62 kJ/kg energy input) to maize germ oil, the phytosterol concentration increased up to 32% after a subsequent incubation time of 24 h. The incubation according to them, increased stress response of the plant “bioreactor”. Similarly, Vallverdú-Queralt *et al.* [15] reported that after application of moderate PEF treatment on tomato juice significantly increased the content of polyphenols than the untreated samples. According to them, the increase could be attributed to the defense response of plants to the moderate intensity PEFs (MIPEF) being applied. MIPEF treatment not only provides potential to induce stress reactions in tomato fruits after 24 h of refrigeration by enhancing metabolic activity and accumulating secondary metabolites, but also can increase permeability of the cellular membrane making the extraction of the bioactive constituent more efficient [15].

However, other samples (S3P2 and S3P3) analysed 24 h after PEF treatments produced less volatiles. This may be due to the lower energy intensities applied to them (0.7–1.0 kV/cm electric field strengths; 34 kJ/kg energy input). In the present study, all volatiles were similar to those reported in Stella cherries [29].

The increasing moderate energy intensities (1.4 to 2.5 kV/cm electric field strengths and 31 to 47 kJ/kg energy inputs) of PEF applied to the samples in this study were able to extract more C_6_ aldehydes and aromatic alcohols. This result is in agreement with the study of Vallverdu-Queralt *et al.* [15]. They reported that low or moderate intensity PEF induced sublethal stress to cells by permeabilizing tissue structures that in turn increased extraction of more volatiles and bioactive constituents. The highest electric field intensity in our study actually corresponded to moderate PEF intensity in other studies of Vallverdu-Queralt *et al.* [15]. The energy intensities used in this study were definitely much lower compared to the intensities used in commercial juice pasteurisation. The intensities used in commercial products are typically about 35–60 kV/cm [19]. In summary, samples with the lowest energy intensities (0.30–1.0 kV/cm, 30–54 kJ/kg) in both S3 and S2 treatments and control samples had the least amount of all the major volatiles.

### 3. Experimental Section 

#### 3.1. Cherry Fruits

Red-fleshed sweet cherries (*Prunus avium* variety Stella) with stalks still attached were harvested by hand-picking between January and early February 2013 at commercial maturity determined by the grower from an orchard located at Alexandra (New Zealand). The cherries were immediately dispatched after harvest and transported overnight to the Food Science Department at University of Otago (Dunedin, New Zealand) using a refrigerated (4 °C) truck. Upon arrival (within 24 h after harvest), the cherries were screened. The cherries with severe and deep flesh damaged and mouldy areas were excluded from the study. The stalks of the cherries were carefully removed while the seeds were pitted (Cherry-It pitter, Progressive, Washington, DC, USA). The remaining edible portion was immediately frozen in liquid nitrogen, vacuum packed in aluminum foil bags (100 grams each) and stored at −20 °C until usage.

#### 3.2. Pulsed Electric Field Treatment

The frozen cherries were randomly taken before PEF treatments. Frozen cherry samples were taken out from the storage room (−20 °C) and prepared by cutting cherries into four cuts, weighing, and then placing them directly into the PEF treatment chamber. No thawing out of the samples was done to prevent juice loss prior to PEF treatments. The chunks were submerged in distilled water (15 °C) at a ratio of 60:40, and then subjected to PEF processing. PEF treatments were carried out using a pilot scale PEF apparatus (Elcrack, HVP 5, DIL, German Institute of Food Technologists, Quakenbruck, Germany) with batch treatment configuration (chamber size of 100 mm length × 80 mm width × 50 mm height). This chamber comprised of two parallel stainless steel electrodes of 5 mm thickness with a separate distance of 80 mm.

For each PEF treatment, a total of 100 g of cherry chunks-water mixture was used. Each PEF treatment combination was carried out in triplicate using three independent batches of cherry chunks-water mixture. The temperature and conductivity of each samples were measured prior to and after PEF treatment, using an electrical conductivity meter (CyberScan CON 11; Eutech Instruments, Singapore). The weight of the samples (cherry chunks and water) was recorded. The PEF operating settings applied were: constant pulse width of 20 μs, different electric field strengths ranging from 0.3 to 2.5 kV/cm, constant pulse frequency of 100 Hz and different pulse numbers ranging from 385 to 10,000. Square wave bipolar pulse shape was monitored on-line with oscilloscope (Model UT2025C, Uni-Trend Group Ltd., Hong Kong, China) during treatment for all samples. 

**Figure 1:**
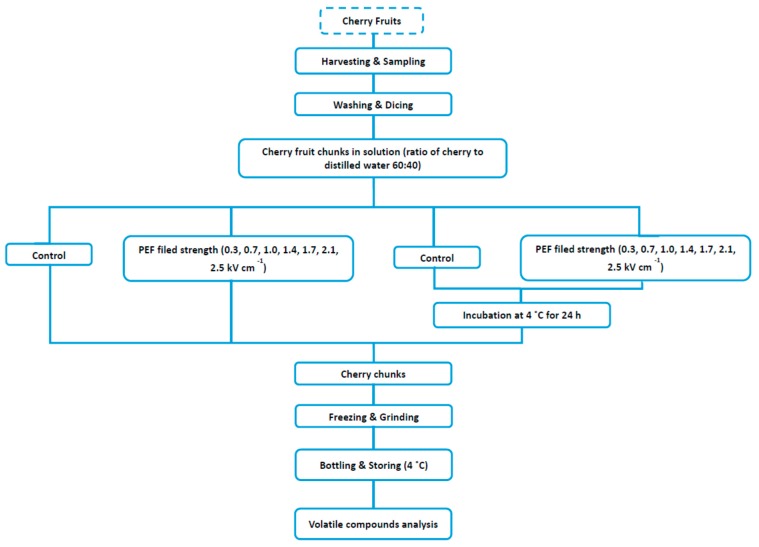
Schematic representation of experimental procedure.

All treatments were conducted at an ambient temperature (~20 °C). In this study, the samples were grouped into three based on storage conditions after PEF treatment. The samples used were: (i) control (untreated) samples before PEF (S1); (ii) samples immediately after PEF (S2); and (iii) samples after PEF treatment and stored for 24 h at 4 °C (S3). Control sample (untreated samples) was also prepared simultaneously with the PEF treated-samples by incubating cherry chunks in water using the same proportion as PEF treated samples. Cherry chunks were separated from the liquid/juice after the processing and storage. All cherry samples were frozen in liquid nitrogen, ground using an analytical grinding mill, and stored at −20 °C prior to to volatile analysis. Schematic representation of the experimental procedure is illustrated in Figure 1. 

Table 2 summarizes the PEF processing settings applied to frozen cherry fruit chunks in solution including sample preparation data and temperature, and conductivity differences. The processing settings applied resulted in effective electric field strengths between 0.3 and 2.5 kV/cm, and pulsed electrical energy between 30 to 55 kJ/kg. These settings ensured that changes in temperature were kept at a minimum and that the final temperature did not exceed 25 °C. The calculation for the specific energy input (W_spec_) generated during PEF treatment of cherry samples was adapted from the equation used in the study of Zhang, Barbosa-Cánovas, and Swanson [35] (Equation (1)):

(1) $$W_{spec}(\text{kJ}/\text{kg})=\frac{VIN\tau }{m}$$

where *V* is the pulse peak voltage in kilo volts (kV), *I* is the electric current in ampere (A), *N* is the number of pulses (dimensionless), τ is the pulse width of square pulses in microsecond (µs), and *m* is the total weight of the sample in kilogram (kg).

The standard deviations reported for the application of the pulsed electric field (Table 2) refer to the instrumental error on repeatability or variability of the processing conditions delivered by the machine to the samples. Repeatability describes the closeness of output readings when the same input is applied repetitively over a short period of time, with the same measurement conditions, same instrument and observer, same location and same conditions of use maintained throughout [36]. The input settings (energy input, pulse frequency, pulse width, pulse number) applied were the same but the indicative readings were different for all replicates.

**Table 2:** Summary of PEF processing conditions and the processing impact on the changes in temperature and conductivity (Mean ± SD).

Sample Codes	Electric Field Strength (kV/cm)	Pulse Voltage (kV)	Pulse Number	Calculated Energy (kJ/kg)	Change in Temperature ^A^ ΔT (°C)	Change in Conductivity of Chunks in Solution
*Samples Immediately after PEF Treatment*						
S2-P1	0.30 ± 0.06	2.83 ± 0.06	10000	39.92 ± 0.10	1.90 ± 0.82 ^a^	1.51 ± 1.03 ^ab^
S2-P2	0.70 ± 0.00	5.50 ± 0.10	6100	54.75 ± 6.16	4.10 ± 1.04 ^a^	1.60 ± 0.51 ^ab^
S2-P3	1.00 ± 0.06	8.33 ± 0.06	2350	48.31 ± 1.37	0.73 ± 0.42 ^a^	0.46 ± 3.30 ^ab^
S2-P4	1.40 ± 0.00	11.10 ± 0.10	900	34.63 ± 1.41	0.47 ± 0.87 ^a^	0.68 ± 1.27 ^b^
S2-P5	1.70 ± 0.06	13.97 ± 0.06	720	43.22 ± 1.06	0.60 ± 1.70 ^a^	0.77 ± 0.74 ^b^
S2-P6	2.10 ± 0.00	13.63 ± 5.92	520	41.86 ± 2.16	0.10 ± 0.52 ^a^	0.12 ± 0.46 ^b^
S2-P7	2.50 ± 0.06	19.60 ± 0.10	385	45.30 ± 1.71	0.33 ± 1.92 ^a^	1.02 ± 1.23 ^b^
*Samples 24 h after PEF Treatment*						
S3-P0	-	-	-	-	Nd	Nd
S3-P1	0.30 ± 0.06	2.77 ± 0.06	10000	29.80 ± 0.09	1.30 ± 1.25 ^a^	3.59 ± 0.53 ^a^
S3-P2	0.70 ± 0.00	5.43 ± 0.06	6100	34.43 ± 3.51	0.87 ± 1.46 ^a^	0.81 ± 0.43 ^b^
S3-P3	1.00 ± 0.06	8.30 ± 0.26	2350	34.26 ± 4.87	0.40 ± 4.35 ^a^	0.26 ± 1.55 ^b^
S3-P4	1.40 ± 0.06	11.47 ± 0.21	900	30.82 ± 2.26	2.10 ± 2.81 ^a^	1.63 ± 0.60 ^b^
S3-P5	1.70 ± 0.06	14.00 ± 0.17	720	42.91 ± 2.13	0.17 ± 1.10 ^a^	1.85 ± 0.49 ^ab^
S3-P6	2.10 ± 0.00	16.73 ± 0.15	520	47.52 ± 2.57	1.23 ± 0.68 ^a^	1.93 ± 0.24 ^ab^
S3-P7	2.50 ± 0.06	19.70 ± 0.30	385	45.46 ± 4.70	1.47 ± 2.90 ^a^	0.76 ± 0.24 ^b^
*Control*						
S1	-	-	-	-	Nd	Nd

Mean ± SD: Standard deviation based on three independent samples and treatments. S1 = control, S2 = PEF, S3 = 24 h incubation at 4 °C after PEF P 0, 1, 2, 3, 4, 5, 6, 7 = 0, 0.3, 0.7, 1.0, 1.4, 1.7, 2.1, 2.5 kV·cm^−1^. ^A^ Changes in temperature due to PEF treatment. The initial temperature of cherry chunks in solution sample prior subjected to PEF treatment averaged at 20.4 ± 0.00 °C. Means within the same column not bearing common superscripts differ (*p* < 0.05; one-way ANOVA with Tukey’s post hoc test). Nd: not determined.

### 3.3. GC-MS Coupled with Automated SPME Analysis of Flavour Volatiles

#### 3.3.1. Extraction of Volatiles using Headspace Solid-Phase Microextraction (HS-SPME)

The volatile extraction in cherry samples using HS-SPME was carried out according to the modified method of Sun, Jiang and Zhao [29]. Ground frozen cherry samples were thawed out at a room temperature, weighed 3.0 ± 0.1 g, and placed in a 20 mL flat bottom headspace vials fitted with a magnetic, PTFE/Silicone red septum and magnetic crimp cap (GERSTEL, Linthicum, MD, USA). Sample in the headspace vial was spiked with 10 μL of thiophene in methanol (0.01 ppm), which was used as an internal standard. To ensure proper distribution of internal standard in the sample, the vial with the sample was mixed using a vortex mixer (Corning, MA, USA). 

For sample preparation, the heads space vial was heated at 35 °C for 5.0 min, using an incubator equipped with an agitator, which was set at a speed of 250 rpm for better extraction. The volatile components in the sample were absorbed onto a gray, notched, 50/30 μm layer of divinylbenzene-carboxen-polydimethylsiloxane (Supelco Co., Bellefonte, PA, USA) fibre on a Stable Flex fiber 23 gauge (OD = 0.63 mm) that was exposed to the sample headspace for 30 min and desorbed for 120 s at 200 °C desorption temperature.

#### 3.3.2. GC-MS Analysis

The Trace GC Ultra (Thermo Scientific, Waltham, MA, USA) GC-MS equipment was used in this study. It was equipped with a DSQ single mass spectrometer, which uses electron impact ionization as ionization source (Thermo Scientific). The GC-MS was installed with a VF-5 ms low bleed/MS fused-silica capillary column (5%-phenyl-95%-dimethylpolysiloxane phase, 30 m × 0.32 mm × 0.50 μm) (Agilent Technologies, Santa Clara, CA, USA). Helium was the carrier gas with a constant flow rate of 1.5 mL/min in the GC-MS. The mode of injection was splitless, and inlet temperature for the injection port was set to 200 °C with 50 mL/min split flow and 2.0 min splitless time. Chromatographic conditions were as follows: the oven was held for 3 min at 35 °C, then raised to 170 °C at a rate of 5 °C/min and held for 2.0 min, then finally heated up to 250 °C and held for 3 min at this temperature. The mass spectrometer was operated in the electron impact mode (EI) with a source temperature of 200 °C, an ionising voltage of 70 eV, and transfer line temperature of 250 °C. The mass spectrometer scanned masses from 50 to 650 *m/z* at a rate of 0.8170 scan/s with a total scan time of 1.22 s.

#### 3.3.3. Multi-Purpose Sampler (MPS)

An automated sample preparation and sample introduction was employed using the Gerstel MultiPurpose Sampler (MPS). This autosampler uses the Gerstel software, which controlled and set the method and sequence of the analysis. The “Prep Ahead” function was used in the analysis wherein the sample preparation steps were performed during the analysis of the preceding sample.

### 3.4. Identification of Volatile Compounds

Peak identification of unknown compounds was carried out by comparison of their mass spectra with spectra in the NIST/EPA/NIH Mass Spectral Database (National Institute of Standards and Technology, Gaithersburg, MD, USA, Version 2.0a, 2002, USA), or NIST web book [37]. To confirm the identity of each volatile compound, retention indices (RIs) were calculated for each volatile compound using the retention times of a homologous series of C_7_ to C_30_ *n*-alkanes (1000 μg/mL in hexane from Supelco), and comparing the RI with compounds analysed under similar conditions in previous literature. The approximate quantities of the volatiles were estimated by comparison of their relative peak areas with that of the thiophene internal standard using a response factor of 1.

### 3.5. Statistical Analysis

The chromatographic data were collated using Microsoft Office Excel 2007 and subjected to statistical analysis using the XLSTAT MX software release 2010 (Addinsoft, New York, NY, USA). One way analysis of variance (ANOVA) was carried out on volatile compounds for each. 

## 4. Conclusions

The PEF treatments applied and storage conditions affected the volatile profile of cherry samples. Three aldehydes and one alcohol (butanal, octanal, 2-octenal, and nonanal, and 2-hexen-1-ol) increased significantly in terms of their concentrations 24 h after PEF treatment at electric field strengths of more than 1.0 kV/cm. Samples incubated for 24 h after PEF treatment (S3) generated higher concentrations of volatiles than samples immediately after PEF treatments (S2). The increase in most volatiles may be attributed not only to the storage conditions but also to the increasing energy intensities applied. Although much higher for S3 samples. 

Findings from this study revealed that more flavour volatiles were released in S3 samples after 24 h storage, and S2 samples immediately after PEF both with the highest electric field intensities. The main volatiles usually found in cherries were shown to be associated with S3 and S2 samples. As expected, control samples (S1 and S3P0) and samples that had been processed with the lowest energy intensities had the least amount and least number of volatiles produced. Overall, the effect of moderate intensity PEF treated samples induced higher amounts of volatile compounds characteristic of cherry flavour. Moreover, no undesirable compounds were detected for all samples because of the low energy intensities applied. The effect of low energy intensity PEF processing of cherries on the bioactive compounds (anthocyanins, polyphenols, vitamin C) will be further investigated. In addition, structural changes, as well as texture, and colour changes are useful quality indicators that determine consumer acceptability.
